# Surface-Induced Nanostructures and Phase Diagrams of ABC Linear Triblock Copolymers under Spherical Confinement: A Self-Consistent Field Theory Simulation

**DOI:** 10.3390/polym10111276

**Published:** 2018-11-16

**Authors:** Ji Wu, Zhihong Huang, Wenchang Lang, Xianghong Wang, Shiben Li

**Affiliations:** 1 Department of Physics, Wenzhou Vocational & Technical College, Wenzhou 325035, Zhejiang, China; kuqikebiag@126.com (J.W.); 13600045268@126.com (Z.H.); wolflwc@163.com (W.L.); 2 Department of Physics, Wenzhou University, Wenzhou 325035, Zhejiang, China

**Keywords:** linear triblock copolymer, self-consistent field theory, spherical confinement, preferential surface

## Abstract

We investigate the nanostructures and phase diagrams of ABC linear triblock copolymers confined in spherical cavities by using real-space self-consistent field theory. Various 3D morphologies, such as spherical concentric lamellae, dumbbell-like cylinder, and rotational structures, are identified in the phase diagrams, which are constructed on the basis of the diameters of spherical cavities and the interaction between the polymers and preferential surfaces. We designate specific monomer-monomer interactions and block compositions, with which the polymers spontaneously form a cylindrical morphology in bulk, and firstly study morphology transformation with a neutral surface when a confining radius progressively increases. We then focus on phase morphologies under the preferential surfaces and consolidate them into phase diagrams. The spherical radius and the degree of preferential interactions can obviously induce the formation of a cylindrical morphology. Theoretical results correspond to an amount of recent experimental observations to a high degree and contribute to synthesising functional materials.

## 1. Introduction

Block copolymers are extensively circulated for their ability to self-assemble and form ordered morphologies at a nanoscale, thereby providing a development platform for fabrication techniques of multifunctional materials. Spontaneous periodic structures possess various potential technological applications in several areas, such as drug delivery, nanolithography, and photonic crystals [1,2,3,4]. Elementary operations of diversifying the formation of block copolymers contain the modifications of molecular architectures, the degree of polymerisation, the volume fractions of components, and the interactions among monomers [5,6], with which the progress of functional materials is insufficient to satisfy the development demands. Various external techniques have been developed in theoretical and experimental studies because the physical morphologies and properties of polymer nanoparticles are not only subject to the internal controlling parameters of polymers but also influenced by external fields and space conditions. For example, hybrid nanomaterials that incorporate inorganic nanoparticles into self-assembled block copolymers under spherical nanoparticle confinement may be used in electronic and optics devices, which can combine the inherent properties of the two units and generate desirable properties [7]. Our work aims to reveal the mechanisms of phase separation and recombination into various nanostructures at the level of molecules.

Studies that have focused on block copolymers in the past decades have laid solid foundations of the skyscraper of polymers. An initial research area, AB diblock copolymer system, has been thoroughly dissected with a simple model and unitary interactions, and typically ordered phases, lamellae, cylinder, body-centred cubic and a gyroid phase are observed in theoretical and experimental studies [5,8,9,10]. An ABC linear triblock copolymer is an upgrade of the diblock copolymer, in which the typical topology can be constructed by sequentially connecting three blocks, that is, a multifold increase in interactions among the blocks. Abetz studied the flow alignment in a large amplitude oscillatory shear field of lamellar block copolymers and observed a totally different orientation behaviour when a third block was introduced to the lamellar AB diblock copolymer [11]. Self-assembled nanostructures are closely related to the topology of polymer chains [12]. Familiar topological structures, including linear, star, cyclic and bottle-brush structures, have been widely studied [13,14,15,16,17,18,19,20,21]. Recent research on the self-assembly of diblock polymer brushes in the spherical confinement of nanoemulsion droplets has found well-defined bicontinuous and axially stacked lamellar morphologies which have opened a new horizon to search for other classical morphologies [22]. Complicated topologies, in which dendritic block copolymers have diversified structures, also exist. An experiment has demonstrated that amphiphilic tree-like macromolecules can form into spherical micelles with unimodal size distributions in an aqueous solution [23].

In bulk geometries, the morphologies of block copolymers are finite, and the properties are immobile; external control methods, such as space confinement, electric and shear field applications, can provide numerous possibilities of polymer design [24,25,26,27,28,29,30]. The principal effects of electric and shear fields are reflected in the formation of long-range ordered nanostructures and directional phase transition [11,31,32,33,34,35,36]. By contrast, the patterns of space confinement are abundant with minimal predictable morphologies. An earlier Monte Carlo (MC) simulation of a cylinder-forming triblock polymer has revealed that the system under thin-film confinement will retain ordered cylinder structures but parallel to the thin film [37]. If the surfaces prefer more than one block, then the cylinder structure would break up. Self-consistent field theory (SCFT) has been implemented on triangular phase diagrams of ABC linear triblock copolymers in a nanopore [15]. The results provide a favourable perspective for observing numerous confined phases arranged as volume fractions and indicate that the composition dependence of morphologies under confinement is more sensitive to the interaction among blocks than to bulk. In a recent small-angle X-ray scattering experiment, special morphologies, such as concentric lamellar, multihelical and spherical-like morphologies, have been observed with different blending ratios in a lamellae-forming polystyrene–block–polydimethylsiloxane/homopolystyrene blend system in cylindrical nanopores [38].

Studies that have concentrated on surface-induced phase transitions have been increasing; however, undiscovered research areas remain given enormous parameter space and multiple confinement models. In contrast to low-dimensional confinement, spherical confinement provides a uniform confining surface; that is, block copolymers inside will be frustrated in all directions. In the present work, we use SCFT to study the frustrated morphologies of a bulk cylinder-forming linear triblock copolymer under spherical confinement. Significant phase behaviour in a variable-sized spherical cavity must be captured. Among many simulation methods, SCFT is applied to investigating polymer chain conformation in phase separation, and a typical advantage is the precise description of the density distribution of each component in an equilibrium state. In addition to the chemically neutral surface, two other preferential adsorption surfaces of the spherical cavity are considered to construct the phase diagrams related to adsorption potential and confinement degree (nanoparticle diameter). The change in free energies of the polymer system for phase transitions is elucidated.

## 2. Materials and Methods

In an SCFT simulation, sophisticated molecular structures are simplified into a coarse-grained model, and complex interactions among copolymer segments are replaced with an average field, which is desired for its high efficiency. We consider a system composed of n ABC linear triblock copolymers confined in incompressible spherical geometries. Each linear triblock copolymer consists of three distinctive chemical compositions. The polymerization index of each chain is specified as *N*, which is set to 400 in our system. The monomeric volumes of the three chemical compositions are assumed to be identical, $v_{A}=v_{B}=v_{C}$, and the statistical segment length of a polymer is denoted as *a*. For convenience, in subsequent equations and diagrams, the lengths and volumes are all expressed in units of mean-square gyration radius, $R_{g}=a\sqrt{N/6}$. A, B and C monomers per chain are expressed as $N_{A}=f_{A}N$, $N_{B}=f_{B}N$, and $N_{C}=\left(1-f_{A}-f_{B}\right)N$, correspondingly. Fredrickson et al. proposed a masking technique to realise thin-film confinement effects [39]; here, we utilise the technique to realise the spherical confinement effects, which are particularly appropriate for pseudo-spectral numerical methods with periodic boundary conditions. The key to realising geometric constraint is introducing an interfacial density function $\phi_{W}\left(\vec{r}\right)$, and a modified incompressibility constraint.

(1) $$\phi_{A}\left(\vec{r}\right)+\phi_{B}\left(\vec{r}\right)+\phi_{C}\left(\vec{r}\right)+\phi_{W}\left(\vec{r}\right)=1,$$

where $\phi_{i}\left(\vec{r}\right)$ represents the volume fraction of species *i*. To enforce the polymer segments into the spherical space, we model a spherical cavity using a cosine function for $\phi_{W}\left(\vec{r}\right)$,

(2) $$\phi_{W}\left(r\right)=\{\begin{array}{cc} 1 & r>R\\ \frac{1}{2}\left\{1+\cos\left[\frac{\pi }{T}\left(R-r\right)\right]\right\} & R-T\leq r\leq R\\ 0 & R-T>r \end{array},$$

where *r* is the distance from the centre of the spherical cavity, *R* is the radius, and *T* is the thickness of the spherical wall. In the piecewise function, $\phi_{W}\left(r\right)=1$ defines the impenetrable hard wall; moreover, $0<\phi_{W}\left(r\right)<1$ represents the blending regions of the surface and copolymer melts, and $\phi_{W}\left(r\right)=0$ represents the regions of full copolymer melts. The confining geometry with the distribution of Equation (2) controlled by the spherical radius *R* can be implemented given a relatively small interface region represented by a small *T*. However, an extremely sharp interface indicates a high spatial resolution and reduces the convergence speed of iteration computational equations. Thus, we select $T=0.45R_{g}$, which is appropriate for our confinement model.

The essence of free energies is the competition between entropy, −*TS*, and internal energy, *U*. In the presence of the interactions between polymers and walls, a sum of the quadratic term must be incorporated into the Helmholtz free energy per polymer chain in a temperature *T*.

(3) $$\begin{array}{cc} \frac{F}{nk_{B}T}=\frac{U}{nk_{B}T}-\frac{S}{nk_{B}}= & -\ln\left(\frac{Q}{V}\right)+\frac{1}{V}\int d\vec{r}\left(\chi_{\mathrm{AB}}N\phi_{A}\phi_{B}+\chi_{\mathrm{BC}}N\phi_{B}\phi_{C}+\chi_{\mathrm{AC}}N\phi_{A}\phi_{C}\right)\;\\ & +\frac{1}{V}\int d\vec{r}\left(\chi_{\mathrm{AW}}N\phi_{A}\phi_{W}+\chi_{\mathrm{BW}}N\phi_{B}\phi_{W}+\chi_{\mathrm{CW}}N\phi_{C}\phi_{W}\right)\;\\ & -\frac{1}{V}\int d\vec{r}\left(\omega_{A}\phi_{A}+\omega_{B}\phi_{B}+\omega_{C}\phi_{C}\right)\;\\ & -\frac{1}{V}\int d\vec{r}\xi \left(1-\phi_{A}-\phi_{B}-\phi_{C}-\phi_{W}\right), \end{array}$$

where $k_{B}$ is the Boltzmann constant; $\chi_{ij}$ is the Flory-Huggins parameter between species *i* and *j*, i, j∈(A,B,C); $\chi_{iW}$ denotes the dimensionless intensity of the interaction between species and preferential surfaces. The local incompressibility constraint in the function is ensured by a Lagrange multiplier, $\xi \left(\vec{r}\right)$. *Q* is the single-chain partition function expressed as $Q=\int d\vec{r}q_{i}\left(\vec{r},s\right)q_{i}^{+}\left(\vec{r},s\right)$, in which the statistical weight function $q\left(\vec{r},s\right)$ denotes a segment of a chain with the free end (*s* = 0) of A block having its *s*th segment at $\vec{r}$. The value of contour variable ranges from *s* = 0 to 1, the terminal of C block. Therefore, the local segment density can be obtained as

(4) $$\phi_{A}\left(\vec{r}\right)=\frac{V}{Q}\int_{0}^{f_{A}}dsq_{A}\left(\vec{r},s\right)q_{A}^{+}\left(\vec{r},s\right),$$

(5) $$\phi_{B}\left(\vec{r}\right)=\frac{V}{Q}\int_{f_{A}}^{f_{A}+f_{B}}dsq_{B}\left(\vec{r},s\right)q_{B}^{+}\left(\vec{r},s\right),$$

and

(6) $$\phi_{C}\left(\vec{r}\right)=\frac{V}{Q}\int_{f_{A}+f_{B}}^{1}dsq_{C}\left(\vec{r},s\right)q_{C}^{+}\left(\vec{r},s\right).$$

The propagator $q\left(\vec{r},s\right)$ satisfies the following differential modified diffusion equations (MDE):

(7) $$\frac{\partial q_{i}\left(\vec{r},s\right)}{\partial s}=\nabla^{2}q_{i}\left(\vec{r},s\right)-\omega_{i}\left(\vec{r}\right)q_{i}\left(\vec{r},s\right),$$

and

(8) $$-\frac{\partial q_{i}^{+}\left(\vec{r},s\right)}{\partial s}=\nabla^{2}q_{i}^{+}\left(\vec{r},s\right)-\omega_{i}\left(\vec{r}\right)q_{i}^{+}\left(\vec{r},s\right),$$

with the initial condition $q\left(\vec{r},0\right)=q^{+}\left(\vec{r},1\right)=1$, where $\omega_{i}\left(\vec{r}\right)$ is the chemical potential field produced by surrounding chains. According to the mean-field approximation, minimising free energy with respect to monomer densities, $\partial F/\partial \phi_{i}=0$, leads to the following expressions for chemical potentials,

(9) $$\omega_{i}\left(\vec{r}\right)=\sum_{i\neq j}\chi_{ij}N\phi_{j}\left(\vec{r}\right)+\chi_{iW}N\phi_{W}+\xi \left(\vec{r}\right),\;i,\;j\in \left(A,B,C\right).$$

The effective volume *V* occupied by copolymers is defined as $V=\int d\vec{r}\left[1-\phi_{W}\left(\vec{r}\right)\right]$ under the spherical confinement, and the effective diameters listed in this study are calculated in accordance with the formula, $V=\frac{1}{6}\pi d_{\mathrm{eff}}^{3}$.

Real-space combinatorial screening method, originally proposed by Drolet and Fredrickson [40], is the general principle for solving the self-consistent Equations (4)–(9). A relax process is assumed by an iteration step, starting with an initially guessed chemical potential field $\omega_{i}$ of species *i*. In accordance with the second-order operator-split method improved by Rasmussen et al. [41,42], the ‘forward’ $q\left(\vec{r},s\right)$ and ‘backward’ $q^{+}\left(\vec{r},s\right)$ end-integrated propagators can be exactly implemented numerically. Here, we use the linear relation of $\omega_{i}^{\mathrm{new}}$ and $\omega_{i}^{\mathrm{new}}$, to realise the constant update,

(10) $$\omega_{i}^{\mathrm{new}}=\omega_{i}^{\mathrm{old}}+\Delta t\left(\frac{\partial F}{\partial \phi_{i}}\right),$$

where Δt=0.1 is effective in solving SCFT calculations in our work. The relaxation steps are repeated until the relative difference among the free energies of the sequential iteration steps is reduced to a satisfactory precision, which is less than $10^{-5}$. The implementation of this process is derived from a minimisation procedure of free energy; this procedure is based on the commensuration between the size of a simulation box and the periodicity of equilibrium morphologies [42,43].

**Figure 1:**
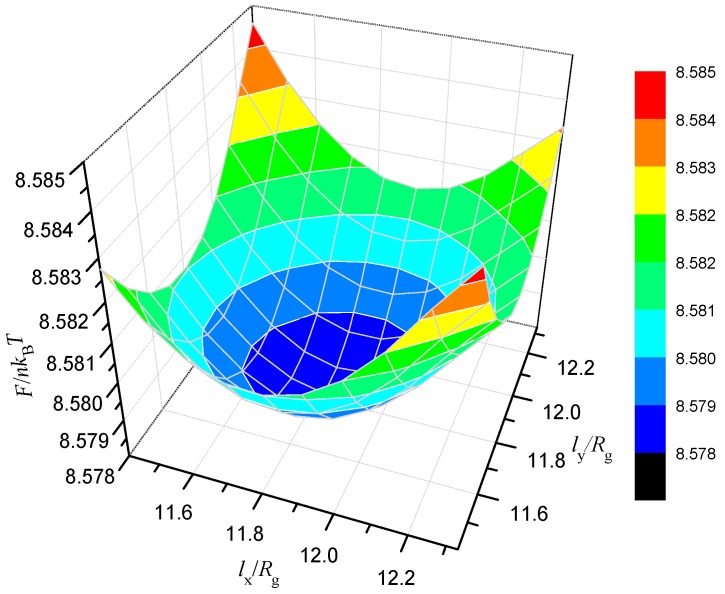
Calculation method for optimising domain sizes in the bulk: optimised domain size for phase point in bulk with $\chi_{ij}N$ = 35 and $f_{A}$:$f_{B}$:$f_{C}$ = 0.2:0.6:0.2; free energy as a function of $l_{x}$ and $l_{y}$, where $l_{x}$ and $l_{y}$ are the sizes of simulation box along the x- and y-directions, correspondingly.

In the present study, the polymers are designed into a rectangular box of $K_{x}\times K_{y}\times K_{z}=64\times 64\times 64$ microlattices with a periodic boundary condition in bulk space, and we adjust the simulation box lengths in two directions to reach stable structures with the lowest free energies. The polymer chain parameters, $f_{A}:f_{B}:f_{C}=0.2:0.6:0.2$ and $\chi_{\mathrm{AB}}N=\chi_{\mathrm{BC}}N=\chi_{\mathrm{AC}}N=35$, are designated throughout the study, and we discover the microphase separation structure at the parameters in bulk by optimising the domain sizes. We adjust the grid spacing in two directions, x- and y-axes, to search for the stable structure, where Δx and Δy vary from 0.178 $R_{g}$ to 0.192 $R_{g}$. Figure 1 illustrates the free energy potential with an equal increment $\Delta =0.0992R_{g}$ in Δx and Δy. A distinct depression area and a rock bottom exist on the free energy surface, which is surrounded by metastable structures. The dimensionless lowest free energy, $F/nk_{B}T$ = 8.578047, locates at the simulation boundary lengths of $l_{x}=l_{y}=11.80R_{g}$. For a spherical confinement, the periodic boundary condition is unnecessary because the spherical diameters directly affect the self-assembled conformations, and the grid spacing is set to $\Delta x=\Delta y=\Delta z=0.15R_{g}$. For the phase behavior with variable spherical surfaces, the diameters and strengths of the surface field are incrementally varied to investigate the significant phases of ABC linear triblock copolymers. We select the most stable phase from all structures produced by proposing different initial guesses of the mean fields by comparing the free energies.

## 3. Results

The phase space of the ABC linear triblock copolymer system within the nanosphere is spanned by four physical parameters, namely, $f_{i}$, $\chi_{ij}N$, $\chi_{iW}N$, and $d_{\mathrm{eff}}$, where *i* = A, B and C. In this study, we concentrate on the influence of confinement, including a subset of the parameter space, $\chi_{iW}N$ and $d_{\mathrm{eff}}$, and designate specific parameters of the copolymer model. We consider symmetric ABC linear polymers, $\chi_{\mathrm{AB}}N=\chi_{\mathrm{BC}}N=\chi_{\mathrm{AC}}N=35$, with which the system is already in an intermediate-separated regime, to facilitate the theoretical results to match the experiment [44,45]. Afterwards, we firstly analyse the phase structure of a symmetric ABC triblock copolymer with the volume fractions of $f_{A}:f_{B}:f_{C}=0.2:0.6:0.2$ in bulk (i.e., without the spherical confinement). On the basis of this foundation structure, we explore the effects of a neutral spherical confinement on a 1D phase diagram by gradually increasing the effective diameter $d_{\mathrm{eff}}$ from 2.60 $R_{g}$ to 8.60 $R_{g}$. Subsequently, the effects of attraction and repulsion between the confinement walls and the block copolymers on the morphology are described on the $\chi_{iW}-d_{\mathrm{eff}}$ plane. For an intelligible measure of preferential adsorption strength, we define a strength proportion $\lambda_{iW}$ in our phase diagrams, $\lambda_{iW}=\chi_{iW}/\chi_{ij}$. To demonstrate the inner structures clearly, the 3D structures are described in red, green and blue, which illustrate the A-, B- and C-rich regions in a space, respectively, even the sectional plots.

### 3.1. Morphologies in Bulk and Phase Diagram under Neutral Spherical Confinement

**Figure 2:**
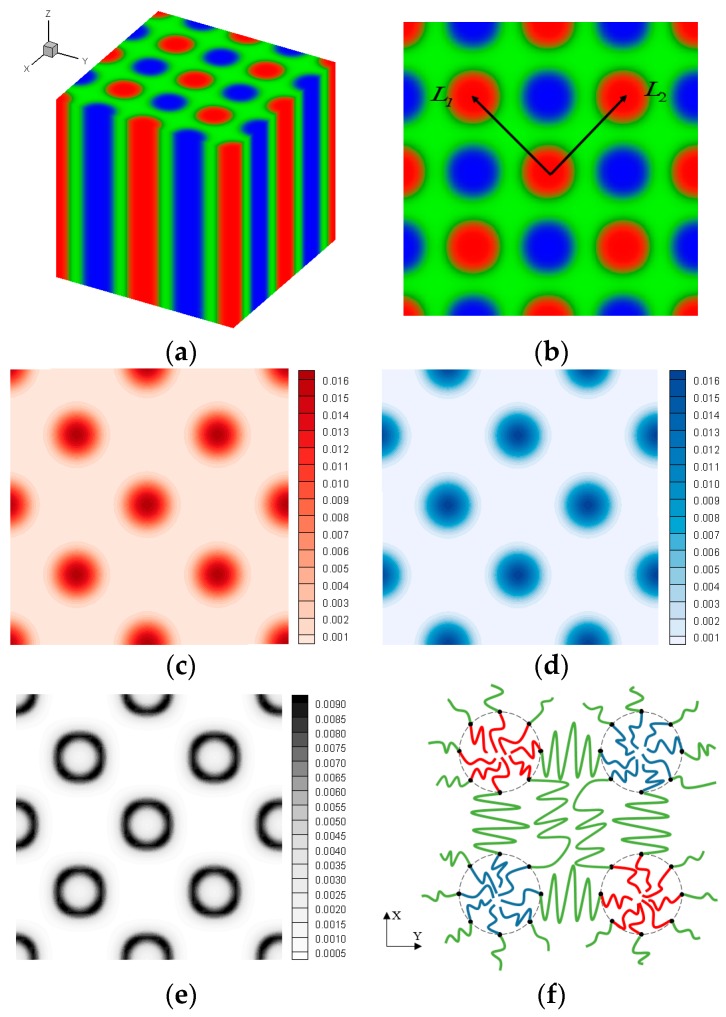
Representative bulk morphology of linear triblock copolymers at $\chi_{ij}N$ = 35 and $f_{A}$:$f_{B}$:$f_{C}$ = 0.2:0.6:0.2. Plots (**a**,**b**) illustrate the 3D two-colour column (TCC) structure and the volume fractions of the system on a plane perpendicular to the columnar axis, where red, green and blue represent A-, B- and C-rich domains, respectively. Density plots for the A-terminal, C-terminal and AB conjunction points on the same plane are depicted in (**c**–**e**), correspondingly. The schematic for a TCC cylinder region is demonstrated in (**f**), in which the grey dashed lines represent the domain interfaces.

In this section, we investigate the effects of the confinement spatial scale on the cylinder-formed ABC linear triblock copolymers with a neutral spherical surface. According to the optimising result in Figure 1, we obtain the two interpenetrating tetragonally arranged cylinder phase (TET_2_) exhibited in Figure 2a, which has been reported in theoretical and experimental studies [45,46,47,48,49]. Plot (b) displays that A- and C-blocks are gathered in the alternate circular regions, whilst outside space is covered with B-block. The results indicate that the periods in two mutually perpendicular orientations, *L*_1_ and *L*_2_, are nearly equal, and the optimal structure is generated when the simulation box is approximately twice the size of the phase period. Perpendicular to the columnar axis, we plot the distributions of the A-terminals, s=0, C- terminals, s=1, and the AB junction points, $s=f_{A}$, in Figure 2c–e, respectively, using red, blue, and black. The plots in (c) and (d) demonstrate that the ends of the A and C blocks all gather near the central regions of the respective rich domain, and the distribution of conjunctions forms a diffused, perfect circle between the A- and B-rich domains, as presented in Figure 2e. In accordance with the segment distributions and orientational ordering, a schematic is presented in Figure 2f for the column-captured domains in the x-y plane, in which B middle block has to bridge them on the dotted lines.

**Figure 3:**
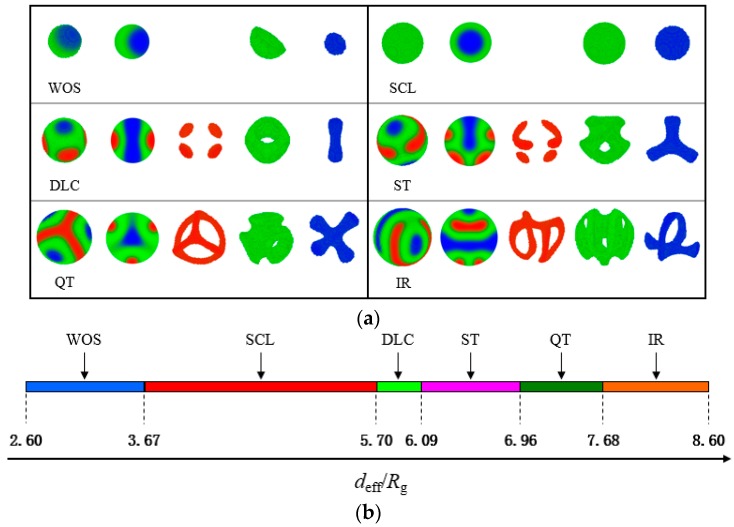
(**a**) Morphologies of the ABC linear triblock copolymers ($\chi_{ij}N$ = 35 and $f_{A}$:$f_{B}$:$f_{C}$ = 0.2:0.6:0.2) confined in a spherical cavity of various diameters (*d*_eff_ ranges from 2.60 *R*_g_ to 8.60 *R*_g_) with neutral surfaces. Red, green and blue represent the A-, B- and C-rich domains, correspondingly. (**b**) Phase stability regions as a function of effective diameter *d*_eff_.

We model a spherical cavity in the centre of the simulation space to study the influence of confined environment on the self-assembled nanostructure and ensure that the polymer molecules cannot escape from the hard wall. The main features of the morphologies of ABC linear triblock copolymers confined in spherical cavities of uniform variable diameters, in which the cavity surfaces are neutral to the confined polymers, are investigated. Six structures are observed under the neutral spherical confinement illustrated in Figure 3a with a proportionable size. These structures are the partially wrapped oblate spheroid (WOS), spherical concentric lamellae (SCL), dumbbell-like cylinder (DLC), semiring-triangle (ST) structure, quadruple texture (QT) structure and irregular ring-like (IR) structure. The structure images listed in each table grid are the overall structure, profile and configuration snapshots of A-, B- and C-blocks, correspondingly.

In the first field depicted in Figure 3a, the WOS structure observed in a small restricted space contains two combined parts, namely, a hollow hemisphere formed by B block and a single solid ellipsoid formed by C block. The hollow hemisphere tightly wraps the solid ellipsoid. On the interface between the explicit regular structure and the confinement wall, even inside the wall, monomers of A block and a fraction of B block are mixed well because of the compression of confinement and the cosine function model for the wall. An interchange of A- and B-rich regions is permissible in view of the complete folded symmetry of the polymer chains, which are the same as the phases shown subsequently. Higuchi et al. reported a similar structure of diblock copolymers under a spherical 3D confinement [50,51,52]. Given that the volume of the confinement space increases and monomers become unbridled, the middle component completely encases a balled-up structure, whilst the other minority component remains hidden. The SCL structure described above is also observed in several other copolymer systems, such as diblock copolymers in spherical nanopores [53]. Onion-like layering systems, each layer being rich in one of the three monomer components, are presented in the study of ABC star triblock copolymers confined in a spherical cavity [54]. The three monomer components all begin to mutually separate, and the rotational symmetric DLC structure occurs when the enclosed space enlarges. An exchangeable minority segment forms a dumbbell standing in the centre of the structure through the entire structure. The other segment discontinuously appears at the equator of the nanoparticle, where the majority middle domain resembles a large cylindrical shell. Subsequently, two small disks of A-monomers are elongated to a semiring shape. The C-monomers form a triangular column domain centred in a B-rich matrix in the ST structure, where the B middle domain resembles a boat anchor. With a continuous increase in a confined volume, the A-monomers are distorted to interconnect and form a coronal structure which resembles a triangular pyramid with arc edges. The C-monomers form a tetragonal column domain centred in a B-rich matrix, in which the latter provides a background for the minority structures to interweave. Overall, the QT structure consists of four identical regions with a threefold rotational symmetry. A normal corona-shell-core structure observed in $A_{2}B_{8}C_{8}$ terpolymer system under rigid spherical confinement is similar to our results [55]. When the spherical diameter $d_{\mathrm{eff}}$ approximates the edge length of the square simulation box $K_{x}\times \Delta x$, an IR structure occurs rather than analogous cylinder structures in bulk.

**Figure 4:**
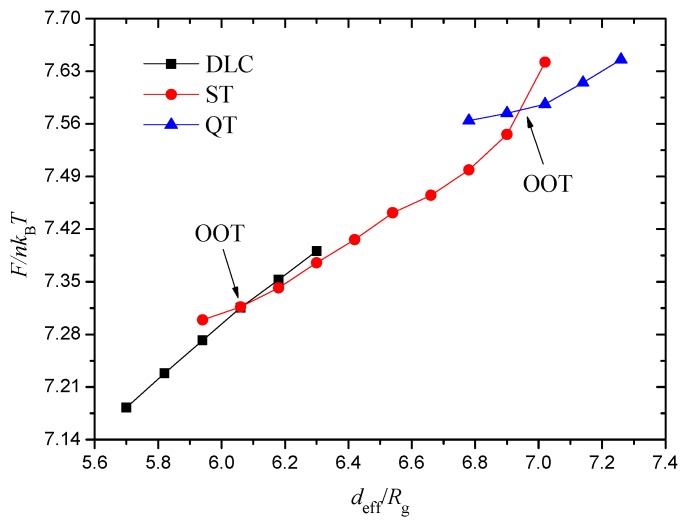
Free energy curves for the three morphologies (dumbbell-like cylinder (DLC), spherical concentric lamellae (SCL), and quadruple texture (QT)) in Figure 3. The order–order transition points are denoted as order–order transition (OOT).

The 1D phase diagram for varying $d_{\mathrm{eff}}$ from 2.60 $R_{g}$ to 8.60 $R_{g}$ is demonstrated in Figure 3b. However, regardless of whether $d_{\mathrm{eff}}$ increases or decreases outside the range, the structures at both ends survive intact, and the data are inconsequential to our work. In calculating the phase diagram, the increment in $d_{\mathrm{eff}}$ is set to 0.12 $R_{g}$ near the phase boundary and is 0.30 $R_{g}$ in other cases. In Figure 3b, with the increase in effective diameter of a spherical cavity, the ordered morphologies described above appear successively. The SCL structure occupies the maximum span, and the DLC structure is briefly present in the diagram. When $d_{\mathrm{eff}}$ is greater than one-half of the edge length $K_{x}\times \Delta x/2$, the ordered morphologies change actively and are complex. We depict the dependence of the free energy $F/nk_{B}T$ as a function of $d_{\mathrm{eff}}/R_{g}$ in Figure 4 to demonstrate that the structures illustrated in Figure 3 transform. The three free energy branches, which correspond to the DLC, ST and QT structures, can be viewed here. The crossing points determine the first-order phase transition among the three studied phases, which precisely testify the phase boundaries exhibited in Figure 3b. A current conclusion of the spherical confinement system is that cylinder-forming linear triblock copolymers assemble into intricate and symmetric structures that are widely divergent with the structures in bulk because the hard wall thwarts the periodicity and applies isotropic compression. Experimentally, there have been some methods to obtain spherical nanoparticles. Zhu and Chen et al. have reported that the polymeric nanorods obtained in anodic aluminium oxide template can transform to spheres upon thermal annealing, which is driven by Rayleigh instability [56,57]. A self-organized precipitation method used to prepare block copolymer nanoparticles can control the particle diameter from several tens of nanometers to several micrometers by changing the preparation conditions, such as concentration of the polymers and evaporation speed of solvent [51].

### 3.2. Morphologies and Phase Diagram in Spherical Cavity with AC-Attractive Surface

In addition to the confinement degree, a short-range interaction between the surfaces and copolymers plays a crucial role in the frustrated packing of compared block copolymers, which has been verified in multiple systems [39,54,58,59,60,61]. Here, we focus on the effects of the confinement degree and preferential surface. On the basis of the 1D research route with the variable $d_{\mathrm{eff}}$, we expand the dimensions of the phase diagram by assuming an alterable interaction between the surfaces and copolymers.

(11) $$-\lambda_{\mathrm{AW}}=\lambda_{\mathrm{BW}}=-\lambda_{\mathrm{CW}}=\lambda \;\left(\lambda \geq 0\right),$$

which represents that A and C components attach to the wall, and the B component is repelled from the surface. The strengths of the three polymer–surface interactions, λ, are constantly kept synchronised in the following study.

**Figure 5:**
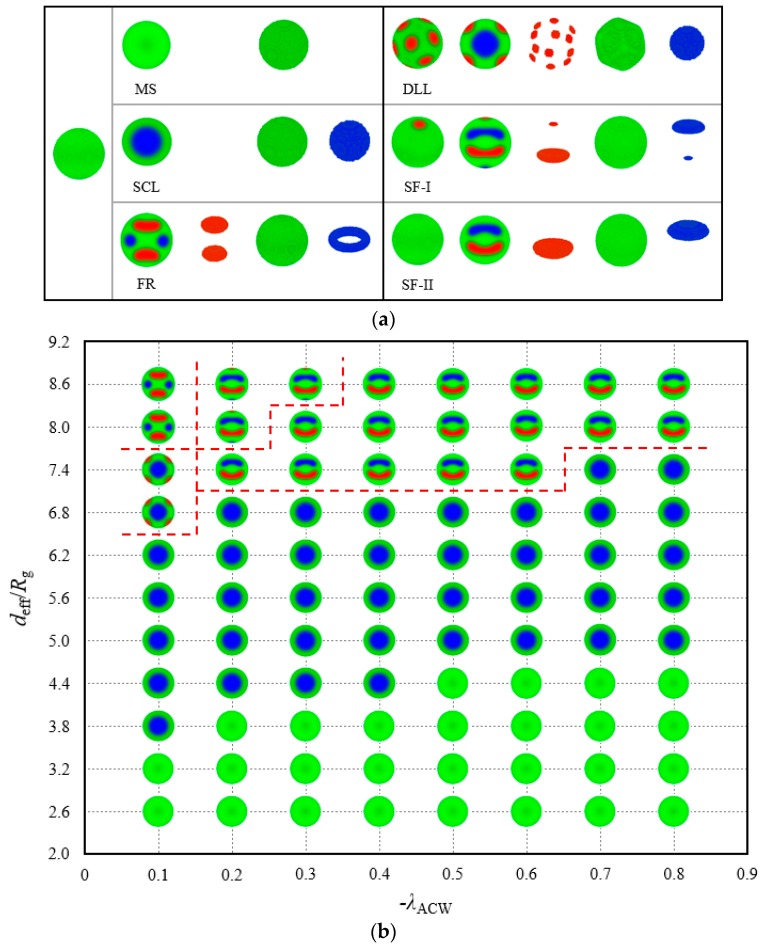
(**a**) Structures of ABC linear triblock copolymers in a spherical cavity of effective diameters, *d*_eff_ = 2.60–8.60 *R*_g_. AC monomers are attracted to the surface, whereas B monomers are repelled from the surface. Red, green and blue represent the A-, B- and C-rich domains, respectively. (**b**) Phase diagram of structures in plot (**a**). The phase diagram is constructed by effective diameters, *d*_eff_ and AC-attractive intensity, $-\lambda_{\mathrm{ACW}}$. The red dashed lines are edge guides for the phase boundaries.

However, a high-precision description of the morphologies in the 2D parameter space is formidable. Considering this case, $-\lambda_{\mathrm{ACW}}$, is increased from 0.1 to 0.8 in an increment of 0.1 and $d_{\mathrm{eff}}$ from 2.6 $R_{g}$ to 8.6 $R_{g}$ in an increment of 0.6 $R_{g}$, thereby producing 88 phase points in the phase diagram. Figure 5a displays six structures with their internal conformations found in the abovementioned parameter array; these structures are the monochromic sphere (MS), SCL structure, Frisbee-ring (FR) structure, discrete lattice-lamellae (DLL), and two types of symmetrical Frisbee (SF-I and SF-II). The MS, SCL and FR structures exhibited in the left column in Figure 5a present the same exterior morphology, similar to homogeneous, which can be distinguished by the profile images. The MS structure closely approximates a homogeneous sphere, except for the mixing region inside the wall, which is removed from the structure images. We call the mixing region a disordered wetting layer, which has been mentioned by Chen and Liang under a thin film system [37]. The A-, B- and C-blocks gather in the wetting layer in contrast to their results given the combined effect of the compression and the attraction from the surfaces. The SCL structure survives from the AC-attractive surface field because of its strong symmetrical stability, thereby indicating that the block chain is insensitive to this polymer–wall interaction. The internal conformation of the FR structure transforms into a layered architecture, which is linked to a relatively large space, as presented in the superstratum in Figure 5b. A minority block forms a ring on the centre plane, whereas the other block forms two Frisbees, symmetrical to each other about the midplane. The FR profile image shows that the concaves of the Frisbees face outward to the wall.

The DLL structure contains one spherical core in the centre and possesses the mirror symmetry. This phenomenon can be considered an evolution from the SCL to the DLL structure with an increase in confinement diameter $d_{\mathrm{eff}}$ under a weak wall–polymer interaction, as illustrated in the column of $-\lambda_{\mathrm{ACW}}=0.1$ in Figure 5b. Several A-monomers break away from the wall and are discretely embedded in the B-block matrix considering the relaxed restriction and weak preferential potential. Moreover, two types of SF structures are observed in the last two columns in Figure 5a. The SF type contains two Frisbees formed by two minority components at the mirror symmetric locations. The profile image clearly demonstrates that the concaves of the Frisbees all face outward to the interior. In contrast to SF-II, a small A-rich disk and a B-rich disk are embedded on the outer layer of SF-I. Overall, A- and C-rich regions appear alternately along the symmetric axis. In particular, these regions have the same formation mechanism and internal interactions but with a large space in the SF-I model. The spherical surface releases a few minority components. In contrast to the case under the neutral surface, these frustrated structures are generally succinct and of notable regularity.

Figure 5b plots the location of these morphologies represented by the shrunken profile images. In Figure 5b, the MS structures are all located at low spherical diameters. The area must be a narrow space that can only accommodate the majority of the middle component and ensure that the middle block bridges on both sides of the wall. The SCL structure occupies the middle area of the phase diagram. A niche for the SCL with any interface interaction λ exists. With a fixed $d_{\mathrm{eff}}$, such as $d_{\mathrm{eff}}=4.4\;R_{g}$, the SCL phase translates to the MS phase with the increase in $-\lambda_{\mathrm{ACW}}$; with a fixed $-\lambda_{\mathrm{ACW}}$, the SCL phase also translates to the MS phase with the decrease in $d_{\mathrm{eff}}$. In this particular parameter environment, the space confinement and surface adsorption field have the same effect on the morphologies. The DLL and FR structures, which correspond to the QT and IR structures in the neutral system, are stabilised only in the upper-left corner of the phase diagram, divided by a red dashed line, of a parameter space ($-\lambda_{\mathrm{ACW}}=0.1$). A reasonable explanation is that the minority components adhere to the spherical cavity walls and then form several layered structures with the fixed linear topological constraint when the adsorption interactions between the walls and blocks are applied. The combination of strong AC-attractive fields and large spherical space leads to the two homologous SF structures that show a high stability in the phase diagram.

**Figure 6:**
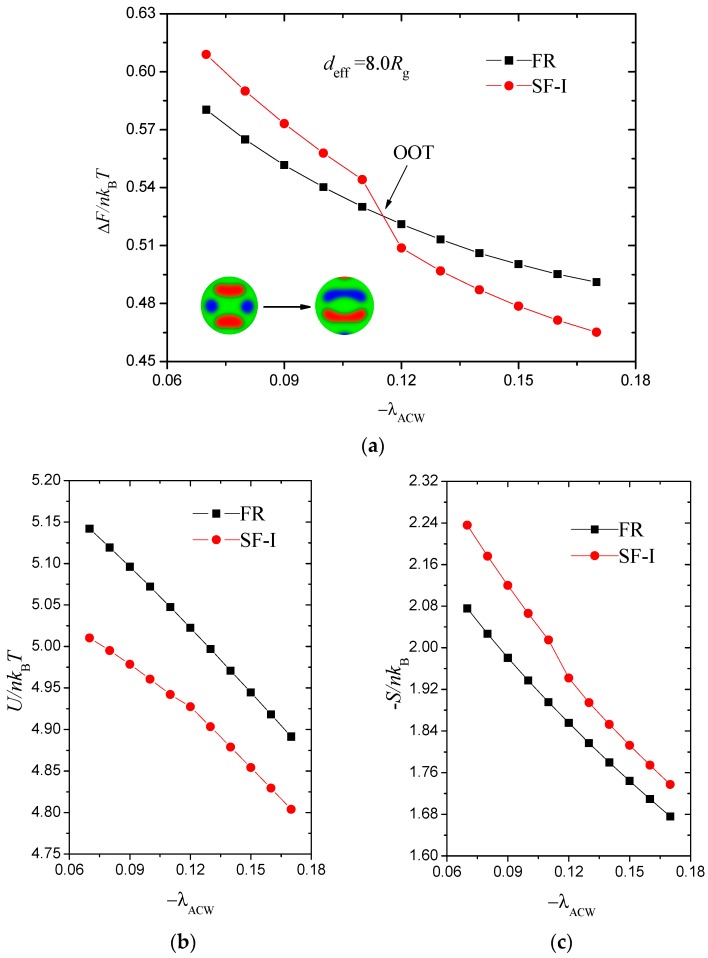
(**a**) Order-order phase transitions with AC-attractive intensity, $-\lambda_{\mathrm{ACW}}$, when *d*_eff_ = 8.0 *R*_g_. We subtract a linear function from the scaled free energies to highlight where the curves cross, and plot $\Delta F/nk_{B}T=F_{\min}/nk_{B}T-\left(7.03-5.61\left|\lambda_{\mathrm{ACW}}\right|\right)$. (**b**,**c**) Internal energy, $U/nk_{B}T$, and entropic energy, $-S/nk_{B}T$, contribute to the free energy in (**a**).

The phase transition between FR and SF phases are subtle; therefore, the transition process from FR to SF-I is obtained along the phase path $d_{\mathrm{eff}}=8.0R_{g}$ as an example. Figure 6a displays the free energies as functions of the AC-attractive strength $-\lambda_{\mathrm{ACW}}$. To highlight the cross, we subtract the free energies from a linear function.

(12) $$\Delta F/nk_{B}T=F_{\min}/nk_{B}T-\left[7.03-5.61\left|\lambda_{\mathrm{ACW}}\right|\right],$$

The two free energy curves are satisfied at approximately $-\lambda_{\mathrm{ACW}}=0.115$, which denotes an order–order transition (OOT). The free energy is higher in the SF-I phase at the $-\lambda_{\mathrm{ACW}}<0.115$ region than in the FR phase, thereby indicating that it is a metastable phase. However, the SF-I phase stabilises at the $-\lambda_{\mathrm{ACW}}>0.115$ region, where its free energy is low. The intersection also suggests that the transition between the FR and SF-I phases is a first-order phase transition. We divide the free energy into two parts, namely, the internal energy $U/nk_{B}T$ and entropic energy $-S/nk_{B}$, to discuss their contribution separately in Figure 6b,c. From a global perspective, $U/nk_{B}T$ and $-S/nk_{B}$ decrease with $-\lambda_{\mathrm{ACW}}$ for both phases. The increase in surface adsorption strength gradually reduces the internal energies and the disordered degree of the system. A sudden drop in the entropic energy of the SF-I phase from $-\lambda_{\mathrm{ACW}}=0.11$ to 0.12 occurs, thus decreasing the free energy. Therefore, the entropic energy is the principal factor behind this phase transition.

### 3.3. Morphologies and Phase Diagram in Spherical Cavity with B-Attractive surface

**Figure 7:**
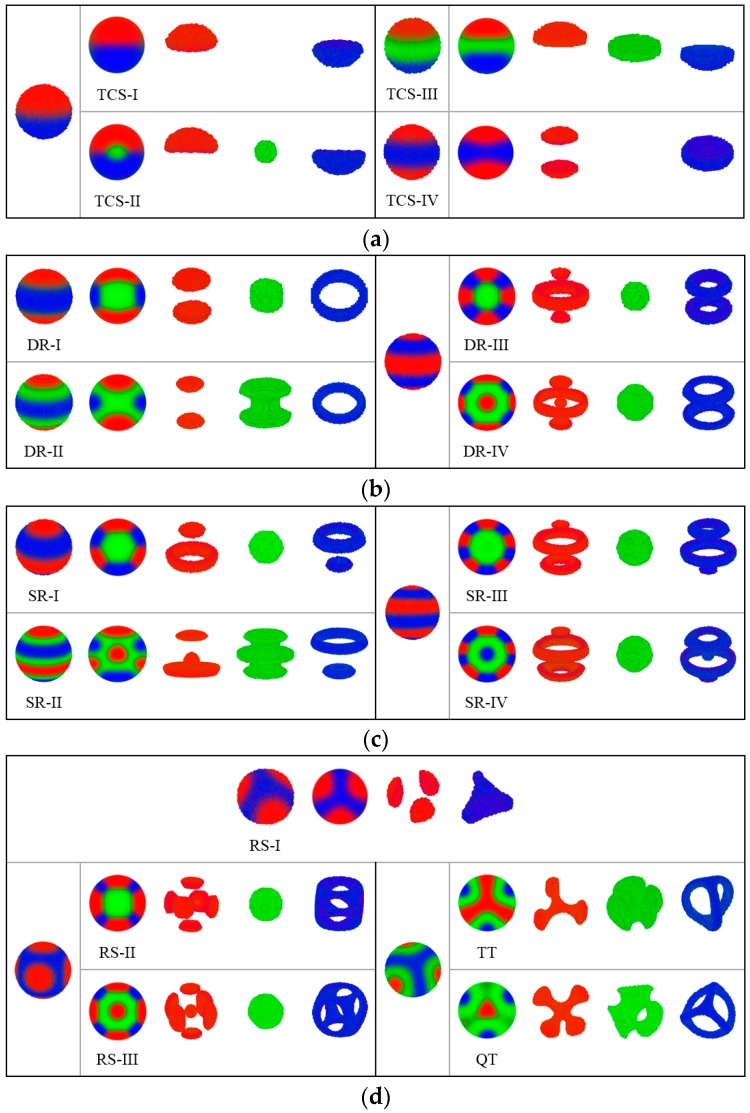
Self-assembled structures of ABC linear triblock copolymers in a spherical cavity of diameters, *d*_eff_ = 2.60–8.60 *R*_g_. B monomers are attracted to the surface, whereas AC monomers are repelled from the surface. Red, green and blue represent the A-, B- and C-rich domains, respectively. (**a**) Two (or three)-coloured sphere (TCS) structures, (**b**) disk ring (DR) structures, (**c**) sphere-ring (SR) structures and (**d**) rotational structure (RS), treble texture (TT), and QT structures.

In this subsection, we examine the inverse adsorption potential, the B-attractive surfaces, to continue the theme:

(13) $$\lambda_{\mathrm{AW}}=-\lambda_{\mathrm{BW}}=\lambda_{\mathrm{CW}}=\lambda \;\left(\lambda \geq 0\right),$$

which represents that the spherical cavity prefers B-monomers and repels A- and C-monomers. Two minority components are no longer constrained but freely interact with the middle component inside the cavity; thus, the structures observed here can be diversified and intricate. On the basis of the structural features and symmetries observed in the 3D patterns, we divide the morphologies into four groups displayed in Figure 7.

(a): Two (or three)-coloured sphere (TCS). Four structures belong to TCS group displayed in Figure 7a. The TCS-I structure is a combination of an A-rich hemisphere and a C-rich hemisphere. In contrast to the MS structure, TCS-I is covered by a B-filled wetting layer. The TCS-II structure replicates the appearance of TCS-I with a small B-rich core that exists in the sphere centre. The core region enlarges and transforms into a disk that separates the A- and C-rich regions in the TCS-III structure. The three TCS structures are different in the B-rich region; however, these structures are homologous and follow the same formation mechanism. Li and Shi et al. observed Janus nanoparticles formed by $A_{8}B_{8}C_{8}$ triblock copolymer confined in small nanopores by using a simulated annealing technique [62]. The Janus nanoparticles have echoes of the formation mechanism from our TCS structures and are considered in single-lamellar structures [63]. TCS-IV contains three disks, of which the C-rich disk is located in the centre region and the A-rich disks on the two sides.

(b): Disk ring (DR). In Figure 7b, from the outward appearance of DR-I, it has the same layered construction as that of TCS-IV. However, C-monomers form a ring instead of a disk and encircle a B-rich cylinder. In a DR-II structure, The B-rich cylinder expands, and several monomers slip the leash from the C-rich ring. In the DR-III structure, the core region contains basically B monomers, the C-rich region forms two rings and the A-rich region forms a large ring accompanied by two small disks. On the basis of DR-III, a new core located inside the B-rich region is formed by A-monomers with the profile map, that is, DR-IV.

(c): Sphere-ring (SR) structure. In Figure 7c, SR-I with axial symmetry includes two identical block domains, in which two disks and two rings formed by A- and C-monomers embed each other. B-monomers gather into a central spheroidal region. When A and C block domains are separated from each other and no longer pack tightly together given environmental change, B-monomers spill out into the interstices, and a minority block forms a solid sphere inside the B-rich region, with which the structure transforms to SR-II. In the SR-III structure, B-monomers form a spherical core, and the A- and C-rich regions form two rings accompanied by a small disk. Compared with SR-I, a four-layer structure turns into a six-layer structure of SR-III, in which each of the two minority blocks forms one more parallel ring. The uniform SR structure can also be assembled by ABC star triblock copolymers with $f_{A}:f_{B}:f_{C}=0.3:0.3:0.4$ in a preferential spherical cavity [54]. An MC study has reported a similar stacked lamella with alternate A and B domains of ABA triblock copolymers under soft confinement [64]. Obviously layered structures are common in a spherical space. SR-IV has the same layered system which contains two disks, that is, two A-rich rings and two C-rich rings with SR-III. The only structural difference is that an additional A- or C-formed sphere occupies the central position of a B-formed core. SR-II and SR-IV structures with newly formed cores are novel discoveries in our study.

(d): Rotational structure (RS), treble texture (TT) structure and QT structure. In Figure 7d, RS-I includes three uniform discrete patches mounted on one large triquetrum core with a threefold rotational symmetry. RS-II and RS-III have the same geometrical framework with a fourfold rotational symmetry. C-block forms a hollow shell with an approximate shape of a cube, in which the central region is filled with B-monomers. A-block forms six patches uniformly inlaying in the surface of the C-formed cube. In addition, an A-rich core exists in RS-III but none in RS-II. In a study of cylinder-forming diblock copolymers, Liang and Shi et al. also found perforated cubic structures [65]. In the TT structure, three arcs formed by a C-block connect the two poles and incise the surface into three identical domains. The A-rich region forms a triangular domain centred in a B-rich matrix with a threefold rotational symmetry. After double checking, we still observe the QT structure as a stable phase under the B-attractive surfaces. In a previous study, virus-like nanoparticles with three and four patches, which correspond to TT and QT structures, have been reported [62]. The overall patchy morphology is largely influenced by the volume fraction of the middle block in linear triblock copolymers.

**Figure 8:**
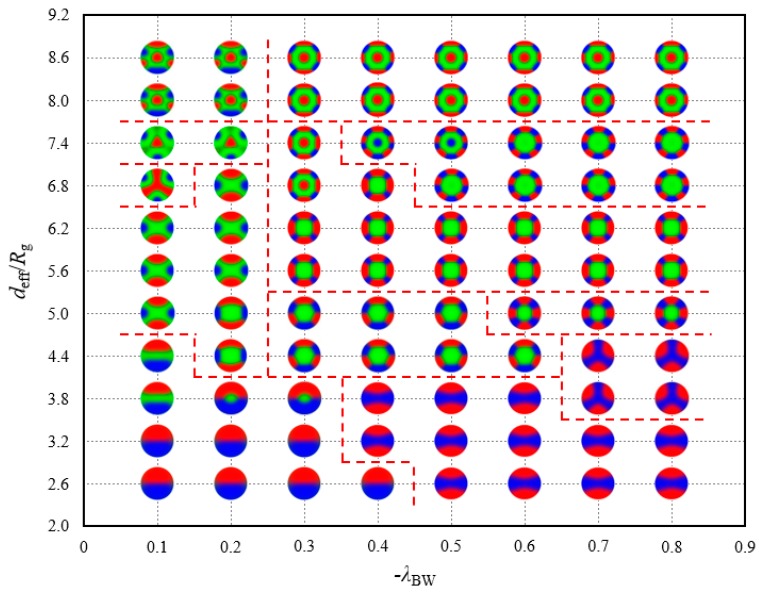
Phase diagram of the structures depicted in Figure 7. The phase diagram is constructed by effective diameters, *d*_eff_ and B-attractive intensity, $-\lambda_{\mathrm{BW}}$. The red dashed lines are edge guides for the phase boundaries.

Figure 8 plots the location of the 17 morphologies which are different from those under the opposite attractive surface field. Among these morphologies, only the QT structure is not destroyed by the B-attractive surface field but is stable in a small phase region, where $d_{\mathrm{eff}}=7.4\;R_{g}$ and $-\lambda_{\mathrm{BW}}<0.3$. For the convenience of observation, the morphologies are represented by the shrunken profile images in the phase diagram. A division by different morphology regions is implemented. Homologous structures, such as TCS-I, II and III, belong to the same area. These structures are presented in the bottom-left region of the phase diagram, in which the spherical volume and adsorption strength are small. With the increase in the strength of the wall preference, TCS-IV is observed in a relatively wide range of $-\lambda_{\mathrm{BW}}\approx 0.4-0.8$ region; with the increase in the diameter of the spherical cavity, DR-I and DR-II are observed in a narrow range of $-\lambda_{\mathrm{BW}}\approx 0.1-0.2$ region. A TT phase point appears between QT and DR-II phase under severe existing conditions, $-\lambda_{\mathrm{BW}}=0.1$ and $d_{\mathrm{eff}}=6.8\;R_{g}$. The two columnar structures, namely, TT and QT, are written in water in our phase space. With the further increase in the spherical volume, QT transforms into SR-II located on the top-left region. SR-I, III, IV, RS-II and III gather in the middle region of the phase diagram. Among these morphologies, SR-I requires a relatively higher confinement degree than others, and SR-III and RS-II remain stable when $-\lambda_{\mathrm{BW}}$ continuously fluctuates upward. However, SR-I cannot bear the powerful adsorption force and has its structure reorganised. A growing $-\lambda_{\mathrm{BW}}$ with $d_{\mathrm{eff}}\approx 3.8-5.0\;R_{g}$ leads to the introduction of RS-I and DR-III. DR-IV is a product of large spherical cavity, in which $d_{\mathrm{eff}}\geq 8.0\;R_{g}$. Not every DR-IV structure observed in our work seems as regular as the illustration in Figure 7b. From an overall perspective, the DR-IV structures obtained when $-\lambda_{\mathrm{BW}}\geq 0.6$ have an alternative five-layer structure; however, the rings are distorted to varying degrees because the large confinement space and strong surface field are comparable to the interaction among blocks. This phase diagram covers numerous complex dynamic processes, which demonstrate that the morphologies of linear triblock copolymers are sensitive to the AC-repulsive surfaces.

**Figure 9:**
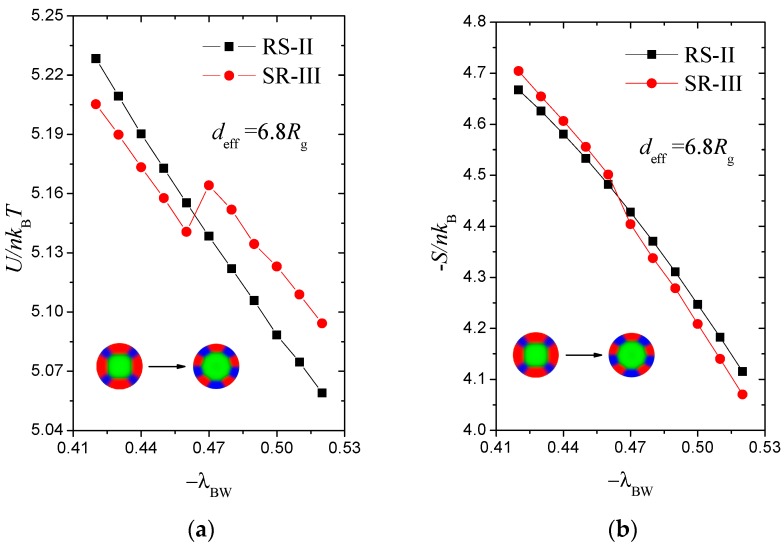
(**a**) Internal energy, $U/nk_{B}T$, as functions of the B-attractive intensity, $-\lambda_{\mathrm{BW}}$, for RS-II and SR-III phases with *d*_eff_ = 6.8 *R*_g_. (**b**) Entropic energy, $-S/nk_{B}T$, that contributes to the free energy for RS-II and SR-III phases.

We study the transition from RS-II to SR-III along the phase path $d_{\mathrm{eff}}=6.8\;R_{g}$ as an example to expose the dynamic process. Figure 9 demonstrates the internal energy $U/nk_{B}T$ and entropic energy $-S/nk_{B}$ as functions of the B-attractive strength $-\lambda_{\mathrm{BW}}$. In Figure 9a, the internal energy U of RS-II decreases progressively, but the internal energy curve of SR-III has an inflection point, which provides favourable RS-II U at a large surface field. By contrast, RS-II has a favourable entropic contribution −S at a small surface field of approximately $-\lambda_{\mathrm{BW}}<0.463$, which dominates the chain configuration, as exhibited in Figure 9b. The preferential surfaces play an important role in the phase behaviour reflected by the variations in internal and entropy energies.

## 4. Discussion

We studied the self-assembly of bulk cylinder-forming ABC triblock copolymers under a spherical confinement by using a real-space SCFT. We detailedly discussed the spontaneous morphologies and phase diagrams on the degree of spherical confinement and the strength of the surface preference characterised by parameters $d_{\mathrm{eff}}$ and $\lambda_{iW}$, correspondingly. The bulk tetragonally arranged a cylinder structure, and the chain conformations were described by analysing its segment distributions. A rigid spherical cavity with a finite thickness was considered, and we positioned the cylinder-forming copolymers to observe its recombination in a neutral surface. We then supplemented varying AC-attractive and B-repulsive potentials on the shield wall and discovered several novel structures obtained in $\lambda -d_{\mathrm{eff}}$ space. Furthermore, we aimed to find several distinctive phase behaviours in an opposite interfacial adsorption potential; in particular, a series of complex and correlative structures with spatial symmetries was demonstrated.

For copolymers under neutral confinement, six atypical phases, namely, WOS, SCL, DLC, ST, QT and IR, were considered stable phases exhibited sequentially in the 1D phase diagram, associated with a changing diameter of a spherical cavity. Systems located at the largest middle range of a length scale in the phase diagram assembled into the SCL structure, thus showing a strong symmetry. The complexity of the internal structure increased with the augmentation of the confinement volume and the frequent transformation of phase structure. On account of the masking technique, the wall thickness might affect the morphologies to a certain extent in our work. On the AC-attractive surface, five new phases, namely, MS, FR, DLL, SF-I and SF-II, were found; these phases had no relation with the cylinder structure. Among these phases, the SF-type phase presented a relatively strong stability in a large spherical space. In addition, the SCL phase remained and filled a large quota in Figure 5b together with the MS phase.

On the AC-repulsive surface, various symmetrical morphologies were demonstrated; among these morphologies, the layered structures were the mainstream. The homologous structures, such as SR-I and SR-II, were derived from the sensitivity of a polymer reconstruction for short-chain-repulsive potential. Several structures, such as DR-III and SR-III, were also confirmed in other polymer systems and external environment. We analysed the free energies and internal and entropic energies of adjacent phases and verified the first-order phase transitions. The influence of preferential surface on entropy energy mainly optimised the phase transitions. Our calculation results would contribute to fabricating advanced polymer materials and could be used for reference in subsequent research.

## References

[1] Zhang S., Chan K.H., Prud’homme R.K., Link A.J. (2012). Synthesis and evaluation of clickable block copolymers for targeted nanoparticle drug delivery. Mol. Pharm..

[2] Register R.A. (2013). Nanolithography: Painting with block copolymers. Nat. Nanotechnol..

[3] Yoon J., Lee W., Thomas E.L. (2005). Self-assembly of block copolymers for photonic-bandgap materials. MRS Bull..

[4] Ma M., Krikorian V., Yu J.H., Thomas E.L., Rutledge G.C. (2006). Electrospun polymer nanofibers with internal periodic structure obtained by microphase separation of cylindrically confined block copolymers. Nano Lett..

[5] Matsen M.W., Schick M. (1994). Stable and unstable phases of a diblock copolymer melt. Phys. Rev. Lett..

[6] Shi A.-C., Li B. (2013). Self-assembly of diblock copolymers under confinement. Soft Matter.

[7] Yan N., Liu H., Zhu Y., Jiang W., Dong Z. (2015). Entropy-driven hierarchical nanostructures from cooperative self-assembly of gold nanoparticles/block copolymers under three-dimensional confinement. Macromolecules.

[8] Matsen M.W., Bates F.S. (1996). Origins of complex self-assembly in block copolymers. Macromolecules.

[9] Matsen M.W. (2002). The standard Gaussian model for block copolymer melts. J. Phys. Condens. Matter.

[10] Khandpur A.K., Förster S., Bates F.S., Hamley I.W., Ryan A.J., Bras W., Almdal K., Mortensen K. (1995). Polyisoprene-polystyrene diblock copolymer phase diagram near the order-disorder transition. Macromolecules.

[11] Stangler S., Abetz V. (2003). Orientation behavior of AB and ABC block copolymers under large amplitude oscillatory shear flow. Rheol. Acta.

[12] Jiang W., Qiang Y., Li W., Qiu F., Shi A.-C. (2018). Effects of chain topology on the self-assembly of AB-type block copolymers. Macromolecules.

[13] Nagpal U., Detcheverry F.A., Nealey P.F., De Pablo J.J. (2011). Morphologies of linear triblock copolymers from Monte Carlo simulations. Macromolecules.

[14] Li W., Qiu F., Shi A.-C. (2012). Emergence and stability of helical superstructures in ABC triblock copolymers. Macromolecules.

[15] Qiu W., He L., Ji Y., Wang X., Li S. (2012). Phase diagrams of ABC linear triblock copolymers under nanopore confinements. Polymer.

[16] Li W., Xu Y., Zhang G., Qiu F., Yang Y., Shi A.-C. (2010). Real-space self-consistent mean-field theory study of ABC star triblock copolymers. J. Chem. Phys..

[17] Li S., Qiu W., Zhang L., Liang H. (2012). Nanostructures and phase diagrams of ABC star triblock copolymers in pore geometries. J. Chem. Phys..

[18] Liu M., Li W., Qiu F. (2013). Segmented helical structures formed by ABC star copolymers in nanopores. J. Chem. Phys..

[19] Atsumi C., Araoka S., Landenberger K.B., Kanazawa A., Nakamura J., Ohtsuki C., Aoshima S., Sugawara-Narutaki A. (2018). Ring-like assembly of silica nanospheres in the presence of amphiphilic block copolymer: Effects of particle size. Langmuir.

[20] Carrillo J.M.Y., Dobrynin A.V. (2010). Molecular dynamics simulations of grafted layers of bottle-brush polyelectrolytes. Langmuir.

[21] Chremos A., Theodorakis P.E. (2014). Morphologies of bottle-brush block copolymers. ACS Macro Lett..

[22] Steinhaus A., Pelras T., Chakroun R., Gröschel A.H., Müllner M. (2018). Self-assembly of diblock molecular polymer brushes in the spherical confinement of nanoemulsion droplets. Macromol. Rapid Commun..

[23] Tian L., Hammond P.T. (2006). Comb-dendritic block copolymers as tree-shaped macromolecular amphiphiles for nanoparticle self-assembly. Chem. Mater..

[24] Ma M., Thomas E.L., Rutledge G.C., Yu B., Li B., Jin Q., Ding D., Shi A.C. (2010). Gyroid-forming diblock copolymers confined in cylindrical geometry: A case of extreme makeover for domain morphology. Macromolecules.

[25] Li W., Liu M., Qiu F., Shi A.-C. (2013). Phase diagram of diblock copolymers confined in thin films. J. Phys. Chem. B.

[26] Hou P., Fan H., Jin Z. (2015). Spiral and mesoporous block polymer nanofibers generated in confined nanochannels. Macromolecules.

[27] Yan L.-T., Schoberth H.G., Böker A. (2010). Lamellar microstructure and dynamic behavior of diblock copolymer/nanoparticle composites under electric fields. Soft Matter.

[28] Ly D.Q., Pinna M., Honda T., Kawakatsu T., Zvelindovsky A.V.M. (2013). Kinetic pathways of sphere-to-cylinder transition in diblock copolymer melt under electric field. J. Chem. Phys..

[29] Pinna M., Zvelindovsky A.V., Todd S., Goldbeck-Wood G. (2006). Cubic phases of block copolymers under shear and electric fields by cell dynamics simulation. I. Spherical phase. J. Chem. Phys..

[30] Jaramillo-Cano D., Formanek M., Likos C.N., Camargo M. (2018). Star block-copolymers in shear flow. J. Phys. Chem. B.

[31] Böker A., Knoll A., Elbs H., Abetz V., Müller A.H.E., Krausch G. (2002). Large scale domain alignment of a block copolymer from solution using electric fields. Macromolecules.

[32] Matsen M.W. (2006). Electric field alignment in thin films of cylinder-forming diblock copolymer. Macromolecules.

[33] Olszowka V., Hund M., Kuntermann V., Scherdel S., Tsarkova L., Böker A., Krausch G. (2006). Large scale alignment of a lamellar block copolymer thin film via electric fields: A time-resolved SFM study. Soft Matter.

[34] Pinna M., Zvelindovsky A.V. (2008). Kinetic pathways of gyroid-to-cylinder transitions in diblock copolymers under external fields: Cell dynamics simulation. Soft Matter.

[35] Pinna M., Schreier L., Zvelindovsky A.V. (2009). Mechanisms of electric-field-induced alignment of block copolymer lamellae. Soft Matter.

[36] Fraser B., Denniston C., Müser M.H. (2005). Diffusion, elasticity, and shear flow in self-assembled block copolymers: A molecular dynamics study. J. Polym. Sci. Part B Polym. Phys..

[37] Chen P., Liang H. (2006). Monte Carlo simulations of cylinder-forming ABC triblock terpolymer thin films. J. Phys. Chem. B.

[38] Cheng M.-H., Hsu Y.-C., Chang C.-W., Ko H.-W., Chung P.-Y., Chen J.-T. (2017). Blending homopolymers for controlling the morphology transitions of block copolymer nanorods confined in cylindrical nanopores. ACS Appl. Mater. Interfaces.

[39] Khanna V., Cochran E.W., Stein G.E., Fredrickson G.H., Kramer E.J., Li X., Wang J., Hahn S.F. (2006). Effect of chain architecture and surface energies on the ordering behavior of lamellar and cylinder forming block copolymers. Macromolecules.

[40] Drolet F., Fredrickson G.H. (1999). Combinatorial screening of complex block copolymer assembly with self-consistent field theory. Phys. Rev. Lett..

[41] Rasmussen K.Ø., Kalosakas G. (2002). Improved numerical algorithm for exploring block copolymer mesophases. J. Polym. Sci. Part B Polym. Phys..

[42] Tzeremes G., Rasmussen K.Ø., Lookman T., Saxena A. (2002). Efficient computation of the structural phase behavior of block copolymers. Phys. Rev. E Stat. Nonlinear Soft Matter Phys..

[43] Bohbot-Raviv Y., Wang Z.-G. (2000). Discovering new ordered phases of block copolymers. Phys. Rev. Lett..

[44] Tang P., Qiu F., Zhang H., Yang Y. (2004). Morphology and phase diagram of complex block copolymers ABC star triblock copolymers. J. Phys. Chem. B.

[45] Tang P., Qiu F., Zhang H., Yang Y. (2004). Morphology and phase diagram of complex block copolymers: ABC linear triblock copolymers. Phys. Rev. E.

[46] Jiang Y., Yan X., Liang H., Shi A.-C. (2005). Effect of polydispersity on the phase diagrams of linear ABC triblock copolymers in two dimensions. J. Phys. Chem. B.

[47] Sun M., Wang P., Qiu F., Tang P., Zhang H., Yang Y. (2008). Morphology and phase diagram of ABC linear triblock copolymers: Parallel real-space self-consistent-field-theory simulation. Phys. Rev. E.

[48] Mogi Y., Kotsuji H., Kaneko Y., Mori K., Matsushita Y., Noda I. (1992). Preparation and morphology of triblock copolymers of the ABC type. Macromolecules.

[49] Mogi Y., Mori K., Matsushita Y., Noda I. (1992). Tricontinuous morphology of triblock copolymers of the ABC type. Macromolecules.

[50] Higuchi T., Pinna M., Zvelindovsky A.V., Jinnai H., Yabu H. (2016). Multipod structures of lamellae-forming diblock copolymers in three-dimensional confinement spaces: Experimental observation and computer simulation. J. Polym. Sci. Part B Polym. Phys..

[51] Higuchi T. (2017). Microphase-separated structures under spherical 3D confinement. Polym. J..

[52] Yabu H., Higuchi T., Jinnai H. (2014). Frustrated phases: Polymeric self-assemblies in a 3D confinement. Soft Matter.

[53] Yu B., Li B., Jin Q., Ding D., Shi A.-C. (2007). Self-assembly of symmetric diblock copolymers confined in spherical nanopores. Macromolecules.

[54] Li S., Jiang Y., Chen J.Z.Y. (2013). Morphologies and phase diagrams of ABC star triblock copolymers confined in a spherical cavity. Soft Matter.

[55] Liu X., Zhou C., Xia H., Zhou Y., Jiang W. (2017). Dissipative particle dynamics simulation on the self-assembly of linear ABC triblock copolymers under rigid spherical confinements. e-Polymers.

[56] Huang Y.-C., Fan P.-W., Lee C.-W., Chu C.-W., Tsai C.-C., Chen J.-T. (2013). Transformation of polymer nanofibers to nanospheres driven by the Rayleigh instability. ACS Appl. Mater. Interfaces.

[57] Xu J., Wang K., Liang R., Yang Y., Zhou H., Xie X., Zhu J. (2015). Structural transformation of diblock copolymer/homopolymer assemblies by tuning cylindrical confinement and interfacial interactions. Langmuir.

[58] Matsen M.W. (1997). Thin films of block copolymer. J. Chem. Phys..

[59] Li W., Wickham R.A. (2009). Influence of the surface field on the self-assembly of a diblock copolymer melt confined in a cylindrical nanopore. Macromolecules.

[60] Han W., Tang P., Li X., Qiu F., Zhang H., Yang Y. (2008). Self-assembly of star ABC triblock copolymer thin films: Self-consistent field theory. J. Phys. Chem. B.

[61] Chen P., Liang H. (2008). Cylinder-forming triblock terpolymer in nanopores: A Monte Carlo simulation study. J. Phys. Chem. B.

[62] Yu B., Deng J., Li B., Shi A.-C. (2014). Patchy nanoparticles self-assembled from linear triblock copolymers under spherical confinement: A simulated annealing study. Soft Matter.

[63] Higuchi T., Tajima A., Motoyoshi K., Yabu H., Shimomura M. (2008). Frustrated phases of block copolymers in nanoparticles. Angew. Chem. Int. Ed..

[64] Sheng Y., An J., Zhu Y. (2015). Self-assembly of ABA triblock copolymers under soft confinement. Chem. Phys..

[65] Chen P., Liang H., Shi A.-C. (2008). Microstructures of a cylinder-forming diblock copolymer under spherical confinement. Macromolecules.

